# Coverage of a Firecracker Blast Injury of the Right Hand With a Chest Wall Flap Under Wide Awake Local Anaesthesia no Tourniquet Technique

**DOI:** 10.7759/cureus.16962

**Published:** 2021-08-06

**Authors:** Alexander S T, Shalimar Abdullah, Parminder Singh Gill Narin Singh, Adzim Poh Yuen Wen, Jamari Sapuan

**Affiliations:** 1 Orthopaedics and Traumatology, Hand and Microsurgery Unit, Faculty of Medicine, Universiti Kebangsaan Malaysia Medical Centre, Kuala Lumpur, MYS; 2 Plastic Surgery, Faculty of Medicine, Universiti Kebangsaan Malaysia Medical Centre, Kuala Lumpur, MYS

**Keywords:** walant, blast injury, lignocaine, adrenaline, covid-19, flap

## Abstract

A flap is done to cover expose structures such as bone, tendon and ligament. Chest wall flaps are usually performed under general anaesthesia due to a fairly large area of surgery and at two different sites which are the chest and the hand. This is the first known reported case of a chest wall flap for coverage of the hand under Wide awake local anaesthesia no tourniquet technique (WALANT). We here report the case of a 32-year-old man who had a firecracker injury over his right hand with bone exposed in his right index and middle finger and distal amputation of the thumb with first carpometacarpal joint dislocation. Chest wall flap reconstruction for coverage of a severe blast injury in the hand is possible and safe under WALANT. The proper technique and administration will lead to a successful surgery without general anesthesia complications and risks. This alternative option may be useful in districts or smaller hospitals where resources are limited.

## Introduction

A flap is done to cover exposed structures such as bone, tendon and ligament [1]. A chest wall flap is not commonly done compared to pedicled flaps or free flaps [2]. Chest wall flaps are usually performed under general anaesthesia due to a fairly large area of surgery and at two different sites which are the chest and the hand. This is the first known reported case of a chest wall flap for coverage of the hand under wide awake local anaesthesia no tourniquet technique (WALANT).

According to Lalonde et al., of 3110 cases done using WALANT in hand and finger surgery, respectively, there were no reported cases of necrosis and infarction [3]. The risk of infarction is close to none as long as the hand and fingers post-debridement is viable with capillary refill times of less than two seconds [4,5]. In other centers, axillary brachial plexus blocks are given combined with general anaesthesia to ensure a secure hand positioning post-operatively [6] but we demonstrated that is this possible with just WALANT.

## Case presentation

A 32-year-old man attempted to throw a firecracker that had rolled under his motorcycle when the firecracker exploded and injured his right hand. This occurred during a local festive season where firecrackers are commonly part of the celebrations. He was presented with the exposed bone of his right index finger till the proximal interphalangeal joint (PIPJ) and exposed bone of his middle finger till the middle part of the middle phalanx. His right thumb had a distal tip amputation at the level of the lunula with an open transverse fracture at the base of the proximal phalanx. He had no other injuries and had no other medical conditions.

**Figure 1:**
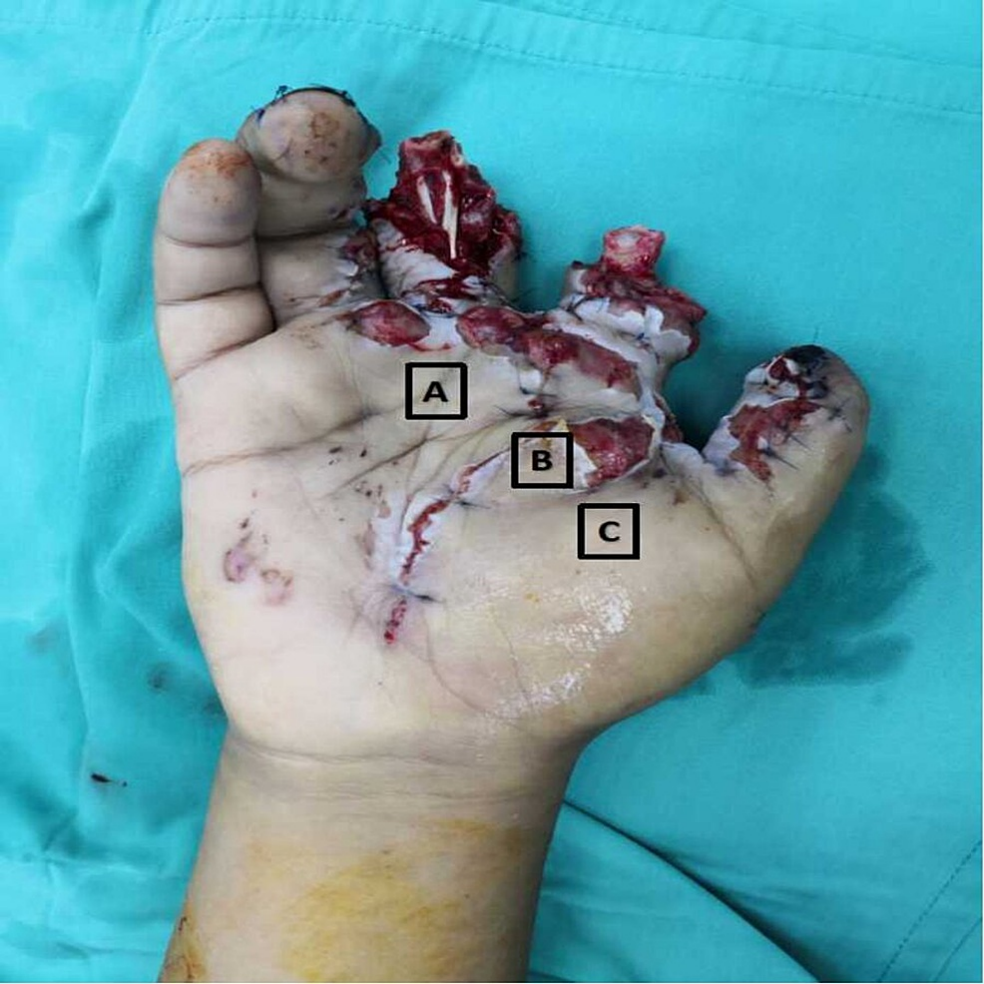
Post-debridement of the firecracker blast injury of the right hand (A) 20 ml of WALANT, (B) 20 ml of WALANT, (C) 10 ml of WALANT

**Figure 2:**
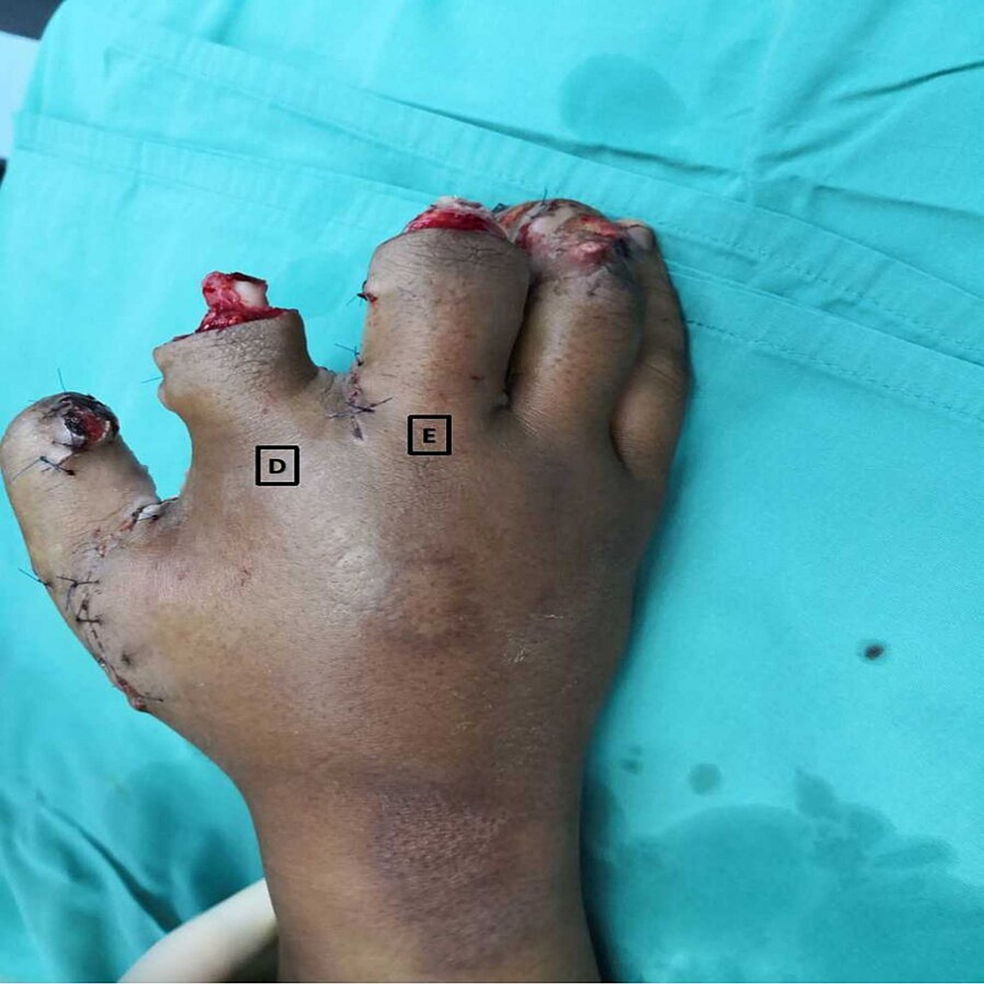
Post-debridement of the firecracker blast injury of the right hand (D) 10 ml of WALANT and (E) 10 ml of WALANT

**Figure 3:**
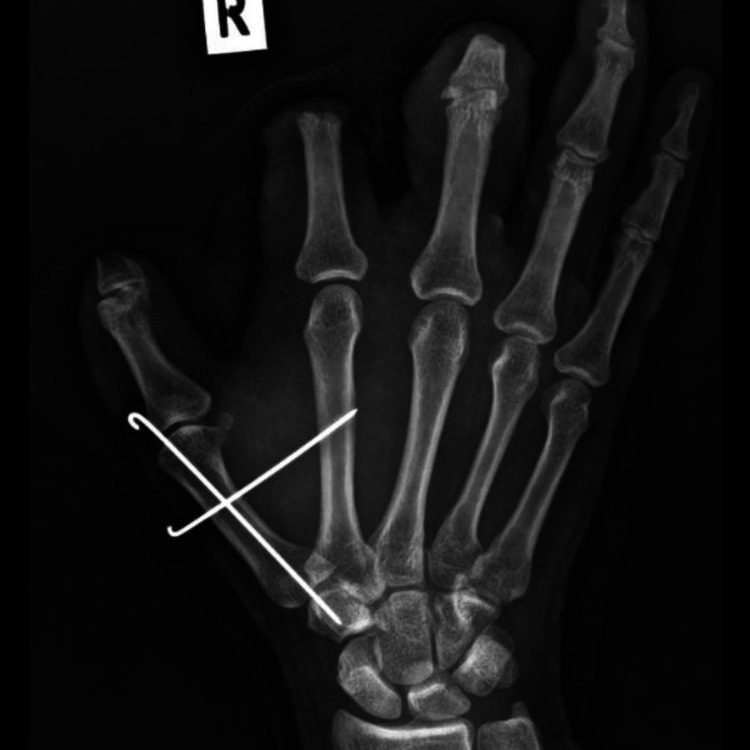
Post-K-wiring of the first carpometacarpal joint (AP view)

**Figure 4:**
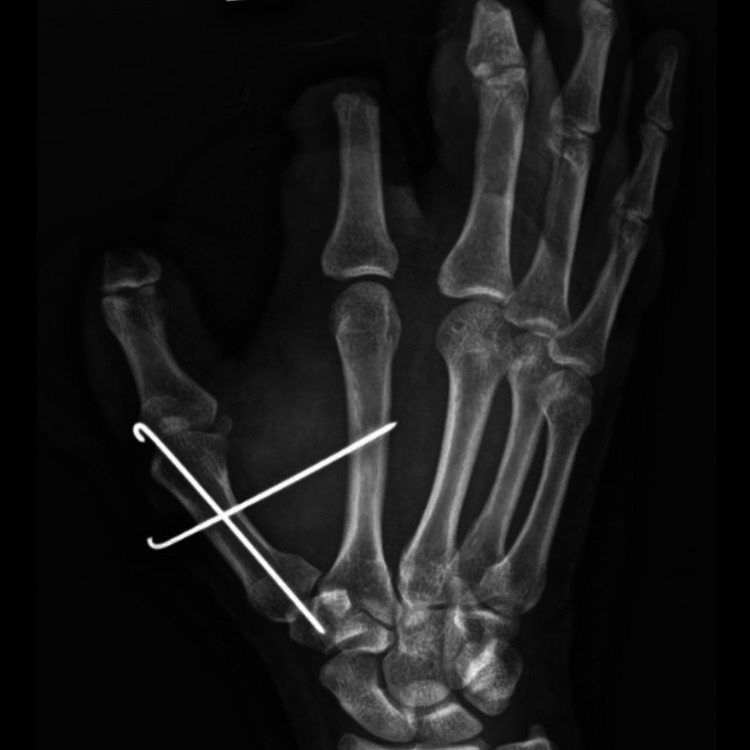
Post-K-wiring of the first carpometacarpal joint (oblique view)

We immediately started him on intravenous cefuroxime and gentamicin. He underwent debridement of the fingers, refashioning of the thumb and K-wiring of the first carpometacarpal joint under general anaesthesia on the same day of injury (Figures 1-4).

On day 5 post-trauma, we preceded with a chest wall flap coverage which was done under WALANT due to limited general anaesthesia availability due to the COVID-19 pandemic.

The WALANT mixture consisted of 50 ml of lignocaine 2%, 40 ml of normal saline 0.9%, 10 ml of sodium bicarbonate 8.4% and 1 ml of 1:1000 noradrenaline [7,8].

**Figure 5:**
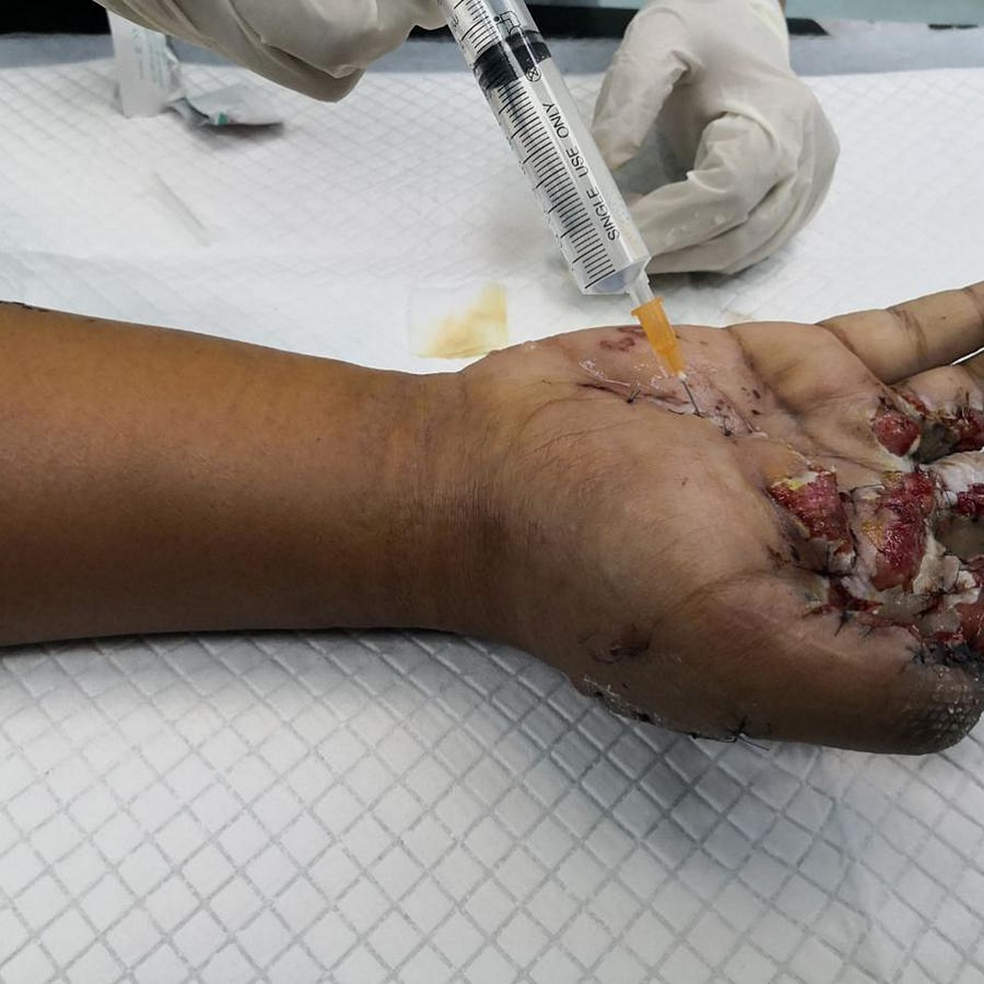
WALANT administration for the right index and middle fingers

The initial injection over the palmar aspect of the hand (Figure 5) had a Visual Analogue Scale (VAS) of 6 out of 10 which we attributed to hypersensitive nerves due to the blast injury. However, this score was reduced to a VAS of 2 out of 10 after infiltration of 10 ml of the solution. Subsequently, to ensure adequate anaesthesia over the thumb, index and middle fingers, we injected a total of 50 ml over the palmar digital nerves thumb (10 ml), index finger (20 ml) and middle fingers (20 ml). We also injected 20 ml of solution over the dorsum of the proximal phalanges (index finger proximal phalanx (10 ml) and middle finger proximal phalanx (10 ml). A total of 80 ml was infiltrated into the right hand.

**Figure 6:**
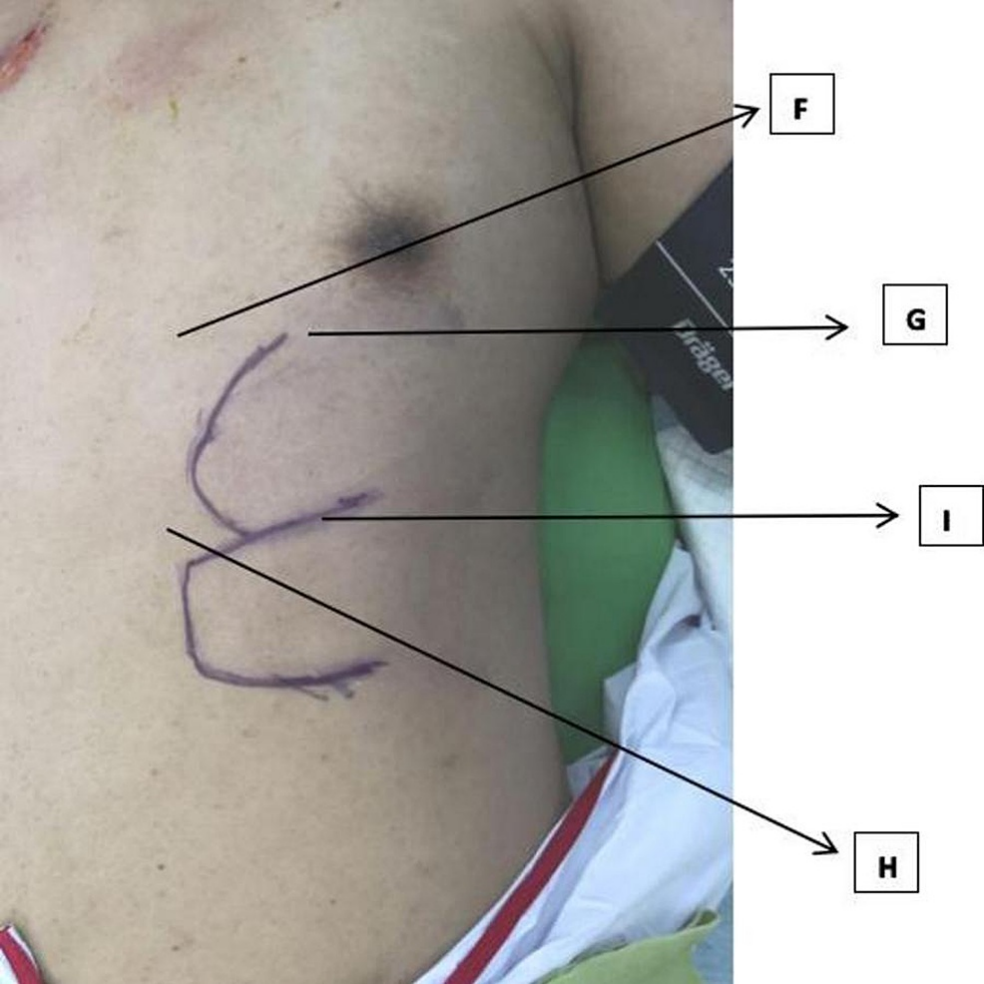
Markings to demarcate surgical incision for the chest wall flap (F)-(I) 10 ml of WALANT at each point

**Figure 7:**
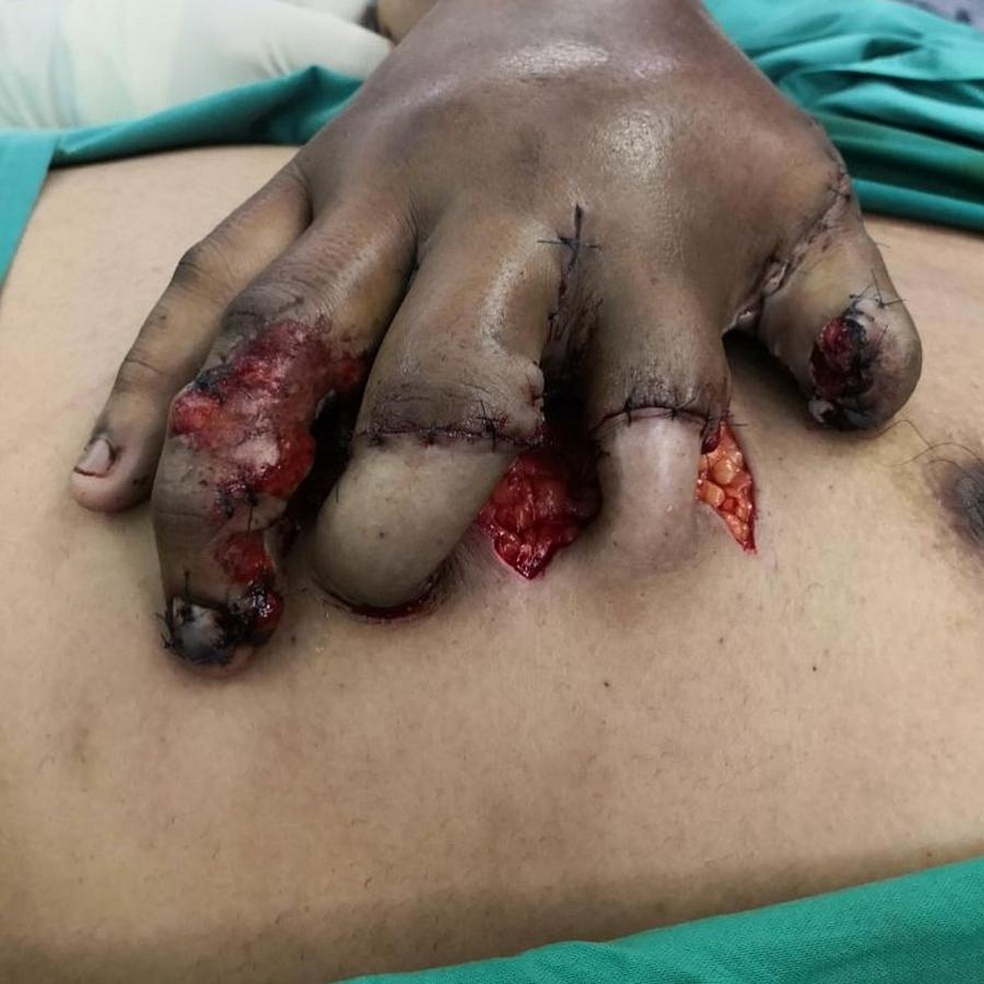
Post-chest wall flap elevation and suturing to the fingers

Over the chest wall, we injected 40 ml of the solution in a rectangular fashion over the marked incision area (Figure 6). Thus, a total of 120cc was used which was ideal for his body weight. His mean intraoperative heart rate was 88 beats per minute (range 80-95) during the surgery. There were no signs and symptoms of lignocaine toxicity. The duration of the surgery lasted 109 minutes (Figure 7).

**Figure 8:**
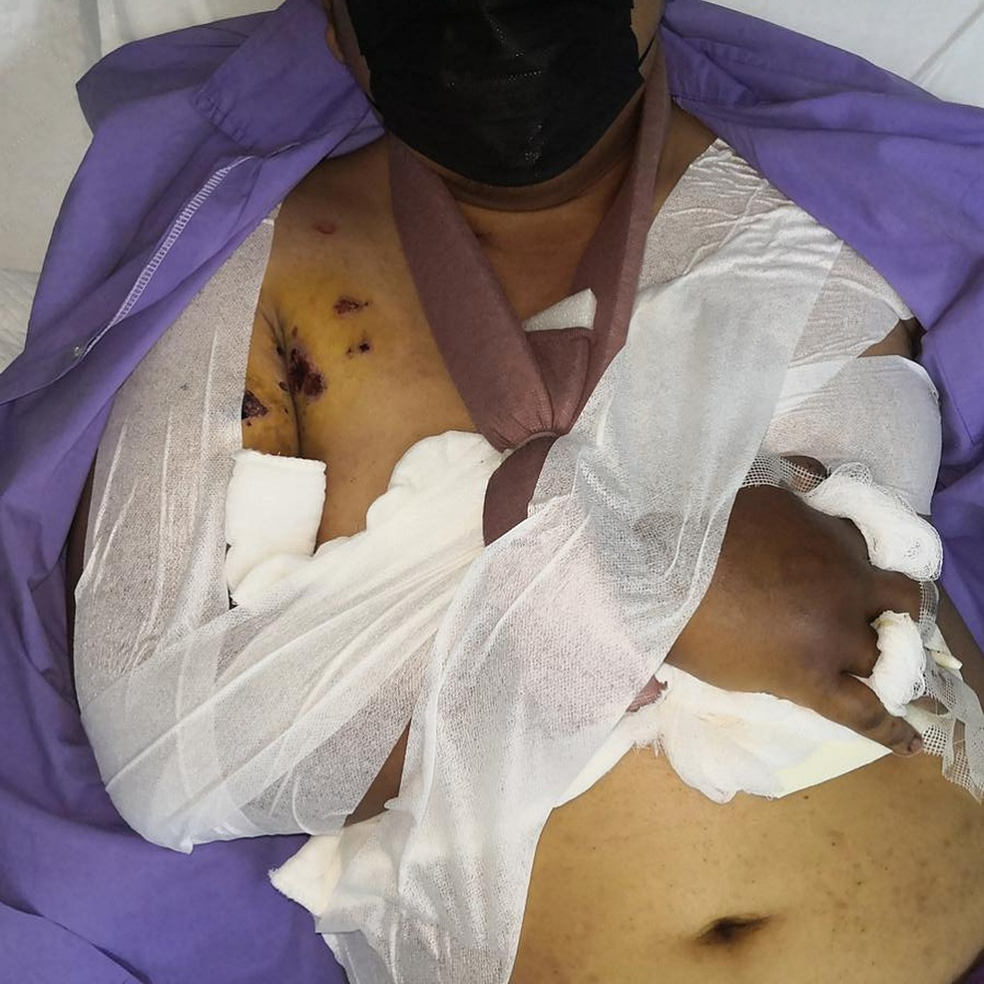
Method of strapping and bandaging of the chest wall flap according to the most comfortable position for the patient

Post-operation, the limb was temporarily immobilized using a collar and cuff with multiple strapping over the shoulder and arm (Figure 8). Multiple large gauze packing was used below his armpit and fold edges to reduce sweat contamination. Daily wound inspection was done and the capillary refill time of the flap was less than two seconds and appeared healthy.

**Figure 9:**
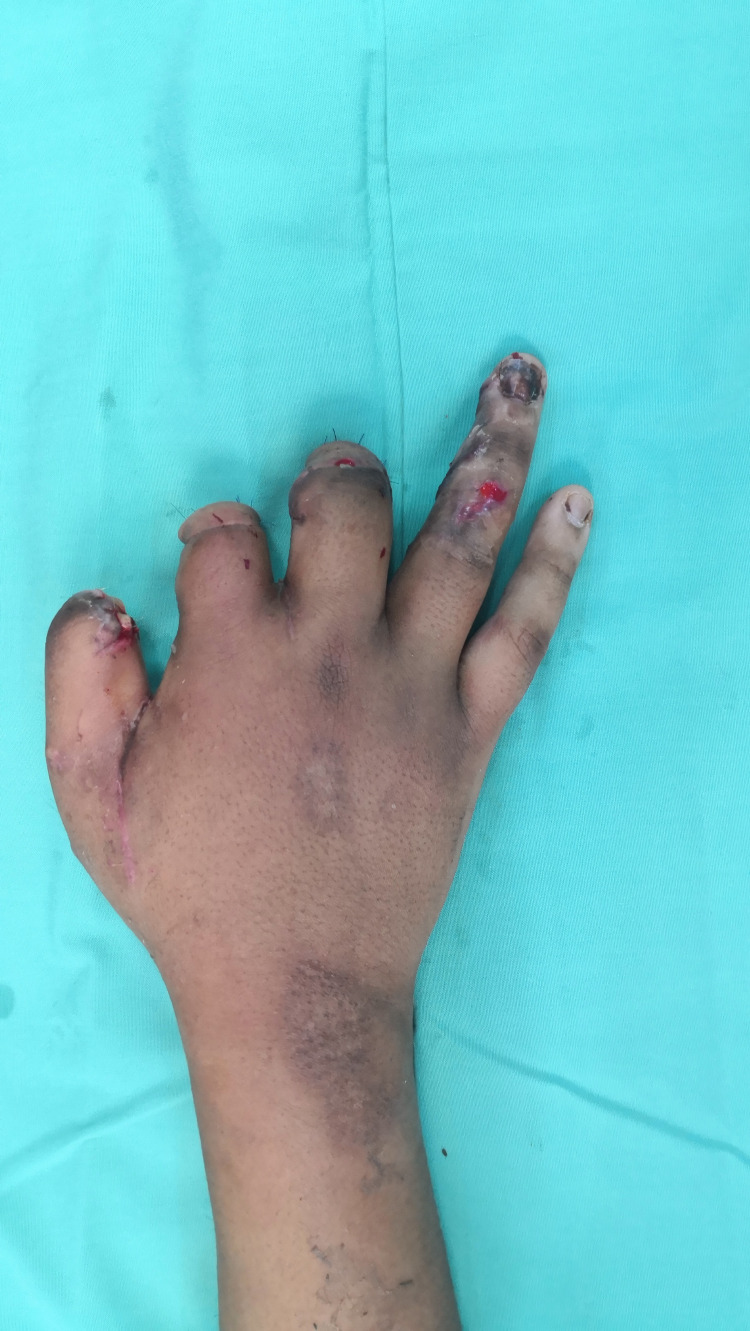
Post-separation of the chest wall flap - dorsal

**Figure 10:**
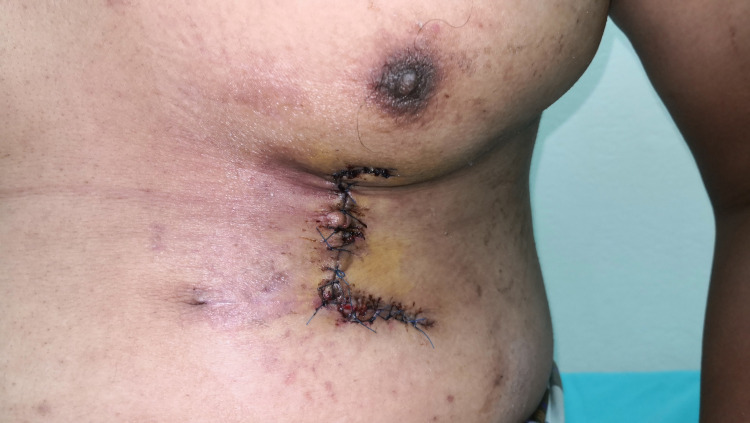
Post-separation of the chest wall flap - chest wall

**Figure 11:**
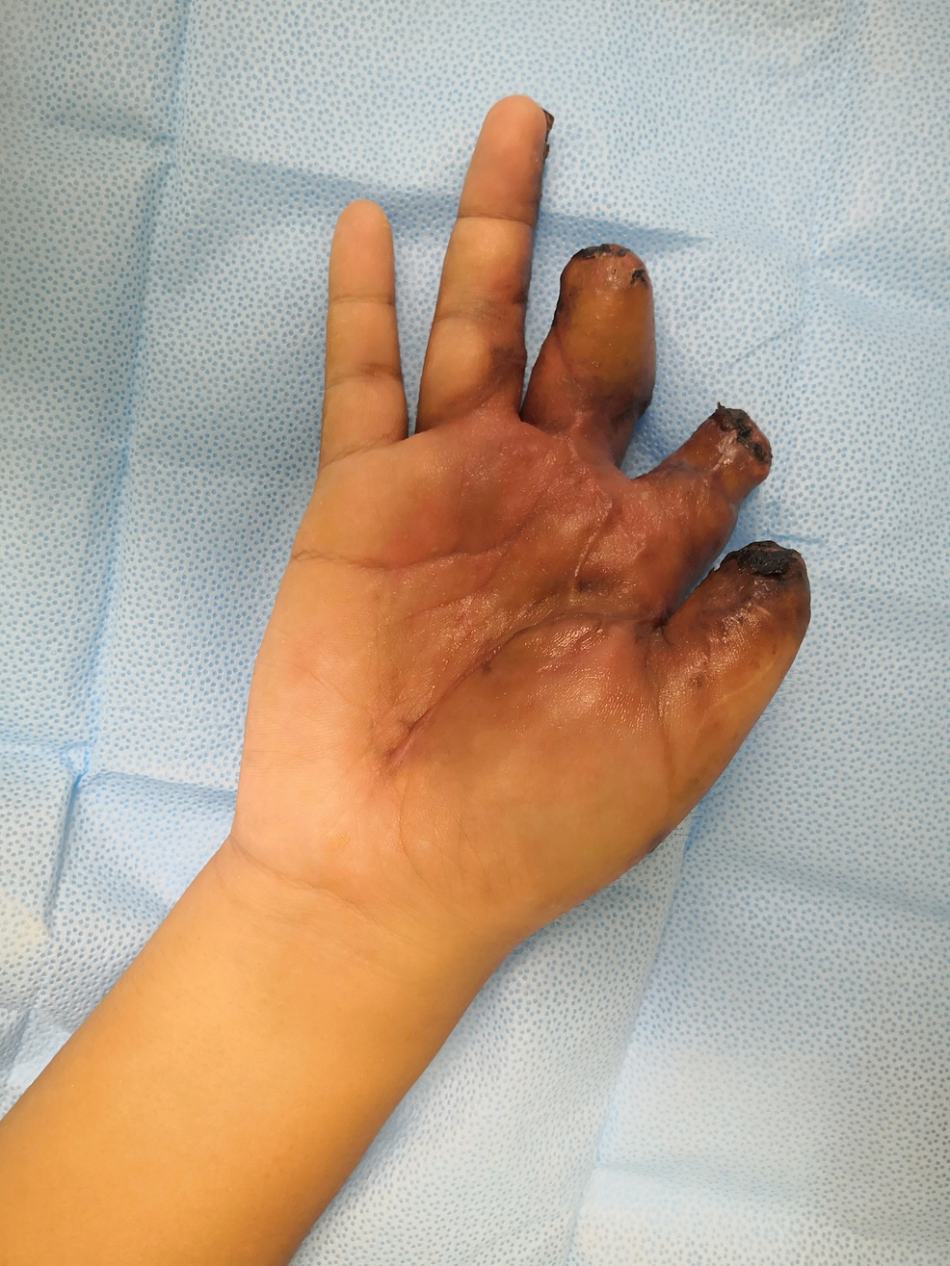
Post-separation of the chest wall flap - volar (three months follow up)

At three weeks after the chest wall flap surgery, we proceeded with the second stage separation of the left chest wall flap with primary closure of the left chest wall donor site. However, as the COVID-19 pandemic had improved for a short period, there was available general anaesthesia time and the surgery was done under general anaesthesia. The right index and middle fingers were detached from the donor site and blanching was observed. The left chest wall donor site was closed primarily with a local advancement flap. The dermal layer was closed with absorbable 3/0 and the skin closed with non-absorbable 4/0 (Figures 9-11). K-wire was removed two months post-op.

Follow up

**Figure 12:**
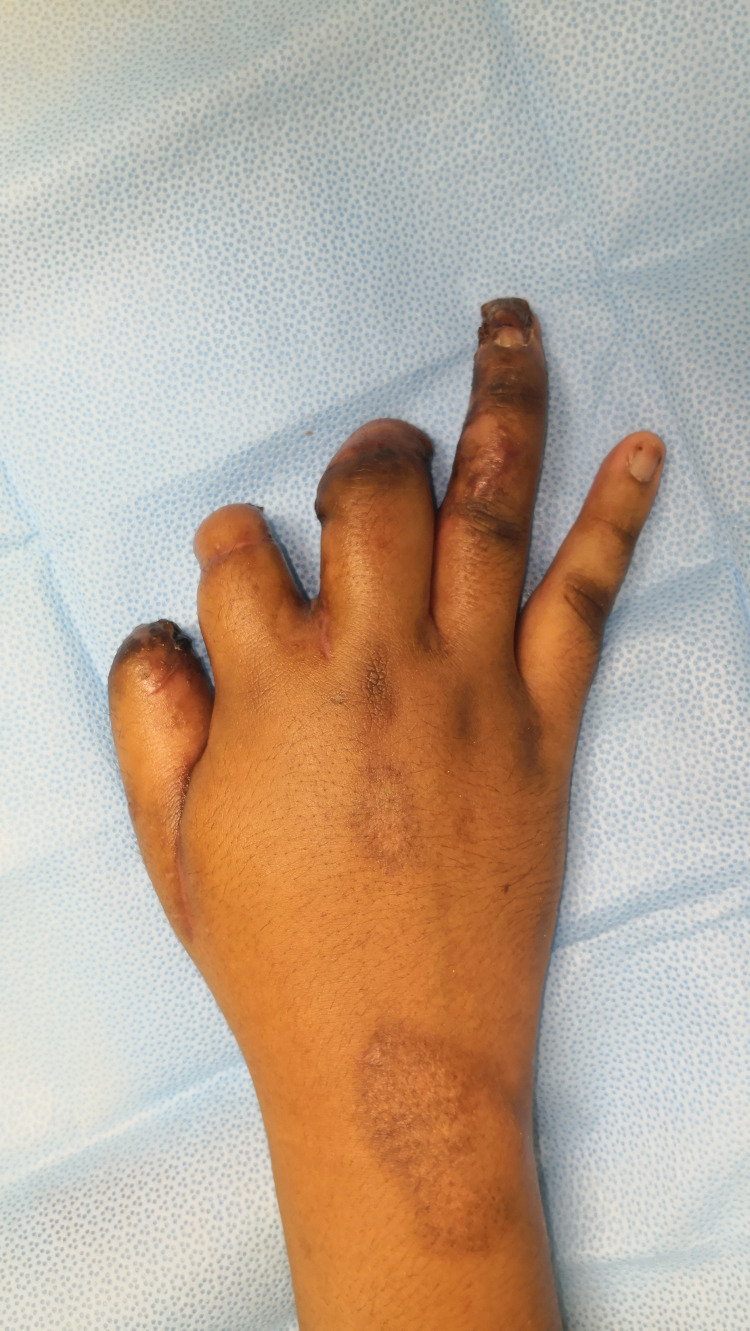
Post-separation of the chest wall flap - dorsal (three months follow up)

**Figure 13:**
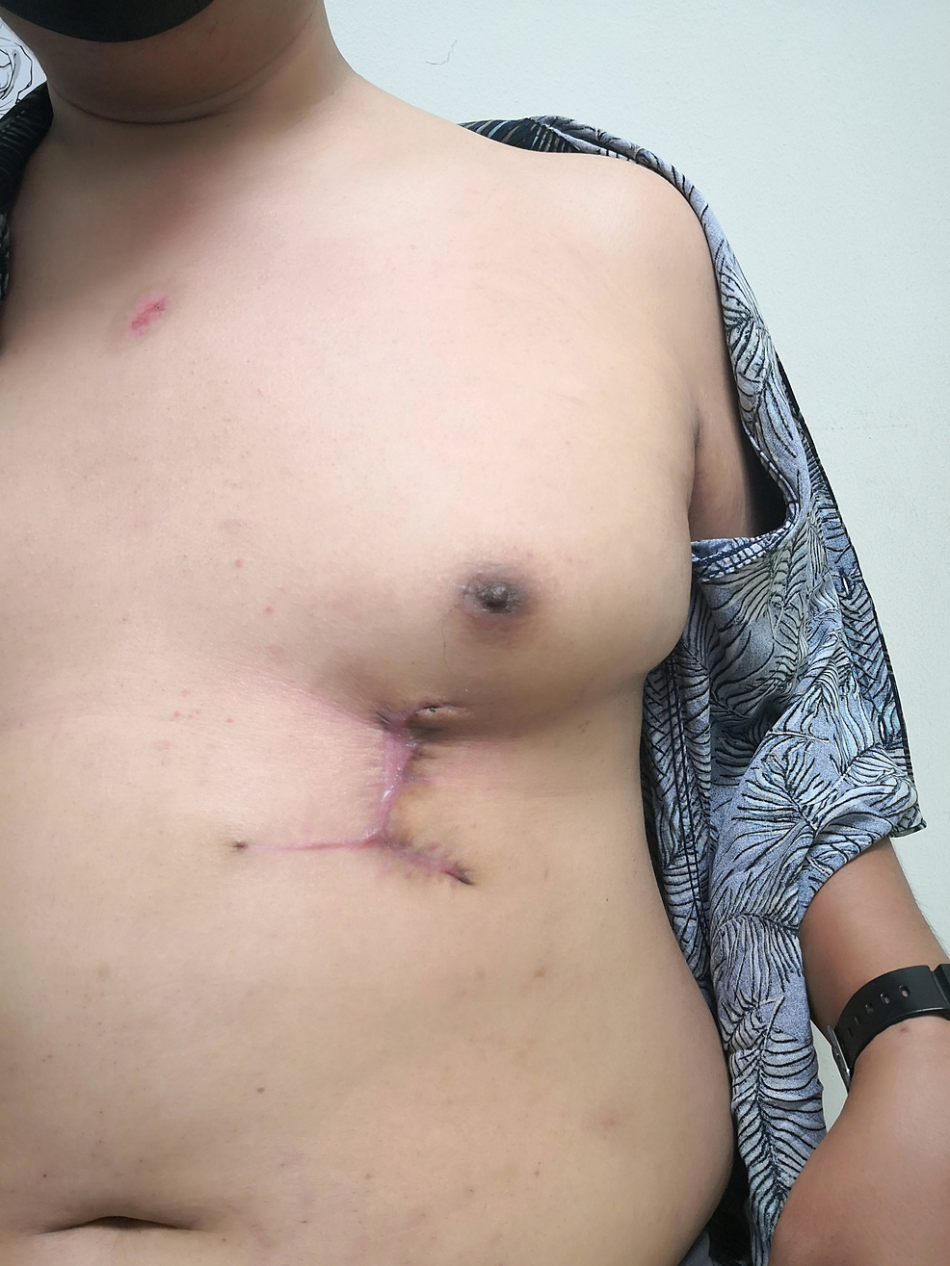
Post-separation of the chest wall flap - chest wall (three months follow up)

He developed hypersensitivity over the palmar aspect, index and middle finger. The index and middle fingertip had no sensation. The 2 point discrimination of the thumb is 9 mm. The radial part of the ring finger is 5 mm and ulna aspect is 7 mm, the little finger radial and ulna aspect is 5 mm. The patient was subjected to physiotherapy to improve his stiffness and to train hand function (Figures 12-13).

## Discussion

In this case, we were able to perform the initial vital first stage of a random pattern chest wall flap under WALANT. This was due to the COVID-19 outbreak with limited general anaesthesia availability.

Early debridement and flap planning is vital for early wound closure and to begin rehabilitation [1]. Furthermore, WALANT is relatively safe and its efficacy is proven. The normal position after the flap reconstruction is to aid the immobilization with adhesive straps [9]. This prevents kinking and aids in the healing of the flap.

WALANT contains adrenaline but did not result in further finger necrosis and ischaemia even in a case of a blast injury. One could argue that the digital arterial vessels may be injured in this situation from the possible reverberations of the blast but this was not the case. The flap bed over the chest wall was also able to heal well without skin infarction despite using WALANT containing noradrenaline and the hand had no failure due to the presence of collateral circulation [5,10]. This patient was successfully done within two stages as compared to other pre-expanded and prefabricated flaps requiring three or more stages which is difficult and time-consuming during a respiratory COVID-19 pandemic [11].

Other possible flap areas would be the abdomen or groin but after discussion with the patient, he preferred the chest wall as he felt it would be the most comfortable position for him. Additionally, the chest wall flap is higher than the abdominal or groin flap regions allowing better oedema control. The patient’s cooperation is vital in preventing flap failure and is able to prevent edema [12]. This could only be achieved without GA through intraoperative counselling thus reinstating the patient's confidence in the surgeon. The use of a flap was able to accelerate the healing over the digits and bone preventing infection and enabled the preservation of the maximum length of the digits [7].

## Conclusions

Chest wall flap reconstruction for coverage of a severe blast injury to the hand is possible and safe under WALANT. The proper technique and administration will lead to a successful surgery without general anaesthesia complications and risks. This alternative option may be useful in districts or smaller hospitals where resources are limited.
